# Assessing Neuro-Systemic & Behavioral Components in the Pathophysiology of Blast-Related Brain Injury

**DOI:** 10.3389/fneur.2013.00186

**Published:** 2013-11-21

**Authors:** Firas Kobeissy, Stefania Mondello, Nihal Tümer, Hale Z. Toklu, Melissa A. Whidden, Nataliya Kirichenko, Zhiqun Zhang, Victor Prima, Walid Yassin, John Anagli, Namas Chandra, Stan Svetlov, Kevin K. W. Wang

**Affiliations:** ^1^Department of Psychiatry, Center of Neuroproteomics & Biomarker Research, University of Florida, Gainesville, FL, USA; ^2^Department of Biochemistry and Molecular Genetics, American University of Beirut Medical Center, Beirut, Lebanon; ^3^Department of Neurosciences, University of Messina, Messina, Italy; ^4^Geriatric Research, Education and Clinical Center, Department of Veterans Affairs Medical Center, University of Florida, Gainesville, FL, USA; ^5^Department of Pharmacology and Therapeutics, University of Florida, Gainesville, FL, USA; ^6^Department of Pharmacology, Marmara University, Istanbul, Turkey; ^7^Department of Kinesiology, West Chester University, West Chester, PA, USA; ^8^Banyan Laboratory, Banyan Biomarkers, Inc., Alachua, FL, USA; ^9^Department of Neuropsychiatry, Kyoto University, Kyoto, Japan; ^10^Department of Biomedical Engineering, New Jersey Institute of Technology, Newark, NJ, USA

**Keywords:** biomarkers, blast injury, brain injury, neurotrauma, blast overpressure, mild TBI, PTSD, neuropsychiatry

## Abstract

Among the U.S. military personnel, blast injury is among the leading causes of brain injury. During the past decade, it has become apparent that even blast injury as a form of mild traumatic brain injury (mTBI) may lead to multiple different adverse outcomes, such as neuropsychiatric symptoms and long-term cognitive disability. Blast injury is characterized by blast overpressure, blast duration, and blast impulse. While the blast injuries of a victim close to the explosion will be severe, majority of victims are usually at a distance leading to milder form described as mild blast TBI (mbTBI). A major feature of mbTBI is its complex manifestation occurring in concert at different organ levels involving systemic, cerebral, neuronal, and neuropsychiatric responses; some of which are shared with other forms of brain trauma such as acute brain injury and other neuropsychiatric disorders such as post-traumatic stress disorder. The pathophysiology of blast injury exposure involves complex cascades of chronic psychological stress, autonomic dysfunction, and neuro/systemic inflammation. These factors render blast injury as an arduous challenge in terms of diagnosis and treatment as well as identification of sensitive and specific biomarkers distinguishing mTBI from other non-TBI pathologies and from neuropsychiatric disorders with similar symptoms. This is due to the “distinct” *but shared* and partially identified biochemical pathways and neuro-histopathological changes that might be linked to behavioral deficits observed. Taken together, this article aims to provide an overview of the current status of the cellular and pathological mechanisms involved in blast overpressure injury and argues for the urgent need to identify potential biomarkers that can hint at the different mechanisms involved.

## Introduction

Traumatic Brain Injury (TBI) represents a major public health problem with an over 150,000 military personnel diagnosed with form of mild traumatic brain injury (mTBI), due to the exposure to blast resulting in a wide range of neurological and psychological symptoms (1, 2). Blast-related brain injuries can be provocatively described as “a silent epidemic of an invisible wound.” Current Explosive mechanisms [improvised explosive devices (IEDs), landmines, and rocket-propelled grenades (RPGs)] are believed to account for 56–78% of Operation Enduring Freedom (OEF), Operation Iraqi Freedom (OIF), and Operation New Dawn (OND) related injuries (3, 4). This has led to labeling the blast-induced TBI (bTBI) as the signature brain injury for combat troops in today’s military (5, 6).

Between 2000 and 2010, the Department of Defense (DoD) reported ~200,000 head injuries as a consequence of combat-related incidents as well as events occurred in a non-deployed environment (civilian injuries) (7). However, even this number may be an underestimate due to the fact that the majority of blast-related mTBIs go misdiagnosed and untreated as a consequence of in-appropriate approaches of screening, invalidated diagnostic criteria or specific detectable abnormalities, and lack of diagnostic tools. Acute blunt penetrating injuries comprised 2.8% of this total, the rest were classified as mTBI (7).

Out of more than 8,000 cases of TBI reviewed by the Defense and Veterans Brain Injury Center, ~50% were related to blast-related barotrauma (8). The clinical features observed in mTBI resulting from blast exposure vary, these include: headache, fatigue, tinnitus, and irritability which have been highly recognized in recent conflicts. Blast overpressure (BOP) injury has been considered the main cause of both morbidity and mortality in neurotrauma (9, 10). Furthermore, blast TBI has been the center for military medical concern in the context of polytrauma, since blast-induced injury, due to its complex components (*primary, secondary, tertiary*, and *quaternary* injuries) is often accompanied by hemorrhagic blood loss, multiple fractures, burns, and systemic injury coupled with TBI (11–13).

The recognition of the high incidence and impact of bTBI; in addition, to the need for a more accurate diagnosis and effective therapeutic interventions, led to an impressive number of experimental and human blast injury studies aiming at investigating the complex interconnected pathways involved in the blast-induced neuropathological/behavioral changes.

This review will focus on three major questions: (i) What is the experimental and human evidence that blast is associated with progressive alterations in the brain and via what mechanism(s) they are mediated? (ii) What is the relation between blast-induced brain injury and the development of neuropsychological disorders such as post-traumatic stress disorder (PTSD)? (iii) What are the biochemical markers that can identify, track and predict the injury and symptoms observed in patients exposed to blast injury?

## Biomechanics of Blast Injury

Blast overpressure-induced injury results from an explosion characterized by an abrupt release of energy in such a short period of time within a small volume creating a non-linear shock and pressure wave (14). The blast shock wave of the primary blast is solitary supersonic pressure wave (peak overpressure) characterized with a rapid (sub-milliseconds–milliseconds) increase in pressure followed by sharp fall in pressure, often to sub-atmospheric levels before returning to ambient pressure (15, 16). This is coupled with the “blast wind” (forced super-heated air flow) that gives rise to a very large volume of gas that may throw victim’s body against other objects. Blast wind, along with the shock wave are the main components of the “blast wave” (17, 18). Blast waves comprise the shock front followed by the blast wind (19). Blast waves impinge on the head-brain complex while mechanical pressure pulses in the brain; the severity of the injury is dependent upon the magnitude and duration of the pressure cycle (20). The net loading at a material point in the brain comprised of a direct transmissive load and deflection-induced indirect loads. The pressure pulse in the brain is governed by the acoustic impedance mismatches between the head and the brain, and the flexural rigidity of the skull (20).

Blast can cause four different types of insults: (i) the *primary injury* resulting from the BOP waves due to the shock-wave overpressure or/and under pressure. This event is usually associated with contusion, edema, hemorrhage, and diffuse axonal injury (DAI) (11, 17, 21, 22). (ii) The *secondary injury* that is due to shrapnel or hard objects propelled at the body. (iii) The *tertiary insult* involves head translation/rotation coupled with acceleration/deceleration due to blunt impact arising from blast wind and finally (iv) the *quaternary insult* resulting from thermal burns or the probable use of toxic gases or chemicals.

Compared to previous past conflicts, the majority of war zone wounds have been attributed to secondary blast injury (shrapnel propelled by explosions), while tertiary and quaternary blast injuries were related to terrorist-linked acts involving structural collapse and the use of toxic material. Previous studies on primary injury (BOP) have traditionally focused on gas-containing hollow organs such as the lungs and gastrointestinal tract (14, 23).

In one study by Clemedson discussing blast injury, the term “blast injury” has been used to describe the biophysical and pathophysiological events post exposure to high explosion or the shock wave associated with it (24). The greatest interest was devoted to study the peak pressure, as well as the impulse relevant to pulmonary injuries produced (25–28). Interestingly, on the pathophysiology focused on the sudden alteration in the body ambient pressure, primarily in gas-air filled organs including the lungs, intestines, or in tissues with different specific weight such as the ear and intestines; this occurred at the interface between media with very large differences in density (16, 24, 29, 30).

Furthermore, BOP can induce a mild form of brain injury with significant neurological conditions involving cerebral edema, neuroinflammation, and vasospasm along with DAI and neuronal death. This neuronal injury phase is followed by a series of complex neuropsychiatric symptoms which may include memory loss and behavioral changes (5, 13, 31–33). As such, exposure to complex blast waves can be viewed as the inducer of multitude of injuries or even polytrauma involving several organ injuries interaction that exacerbates blast insult outcome (13). Finally, blast wave propagation to the brain parenchyma is another controversial mechanism which may involves both direct propagation through the skull or in an indirect propagation via blood vessels which has a direct implication on vascular disturbance (31, 34).

Blast wind passage to the skull causes acceleration/rotation to the brain comprising the direct injury. Indirect injury involves the compression of the abdomen and chest transferring kinetic energy to the body’s biofluid. This rippling effect generates oscillating waves from blood to the brain distant from the contact point. In turn, this kinetic energy transfer will induce functional and morphological changes in brain structures which represent a distinct complex feature of blast-induced brain injury not present in other traditional brain injury models (21, 31, 35). The complex mechanism of blast injury involves consequences of primary blast effects on autonomous nervous system. Taken together, it should be comprehended that the mechanics of neurotrauma due to blast injuries are quite different from that of other types of injuries arising from motor vehicle accidents (blunt) or penetrating injuries (ballistics).

## Neuropathological Alteration in Blast Injury

Experimental studies of primary blast brain injuries (though limited) have shown evidence of altered cellular, molecular and biochemical processes, and behavioral outcomes. For instance, different studies have shown a heterogeneous profile of brain-associated cellular impairments including: elevation in β-amyloid precursor protein, altered expression of protooncogenes *c-Myc, c-Fos*, and *c-Jun* and impaired axonal transport along with oxidative stress with elevated nitric oxide generation (8, 33, 36–44). In addition, neuronal injury and glial activation (discussed later) coupled with elevation of biochemical markers such as, neuron specific enolase (NSE), ubiquitin C-terminal hydrolase 1 (UCH-L1), and glial fibrillary acidic protein (GFAP) have been also reported. Other studies have shown evidence of axonopathy, edema, and hypertrophic astrogliosis with pronounced altered gene expression post-injury event (40, 44–46). However, there were a lot of ambiguity in the overpressure and duration utilized and the methods used to measure these parameters which were often unclear and not standardized (33, 43, 47).

Furthermore, such heterogeneous neural profile has been attributed to several factors including the suitable experimental model systems that can closely mimic “composite” primary, secondary, tertiary, and quaternary components of blast exposure, the lack of standardized blast wave instruments, different body localization and body armor, and the use of different animal species (31, 32, 41, 48) (see Table 1).

**Table 1:** **Recent major studies on experimental blast injury with different parameters assessed (behavioral, neuropathological, and biomarker changes)**.

Reference	Animal model/device used-BOP intensity	Time point assessment post injury	Repeats of blast and time between exposure	Additional variables studied	Behavioral assessment (if available)	Neuro, systemic, and other organ-specific pathology/biomarkers parameters
Abdul-Muneer et al. (102)	Rat/primary blast/shock tube/123 kPa	1/6/6/24/48 h/8 days	One or two (24 h between intervals)	None		Vascular damage, BBB leakage, neuroinflammation MMPs changes, AQP-4, oxidative stress (4HNE-3-NT), and edema; S100B and NSE (serum)
Ahmed et al. (136)	Rat/compressed air-driven shock tube/138 kPa	1, 3, 7, 14, 26, 36, and 42 days	Single or five (24 h between each blast)	Repeated vs. single blast comparison		Oxidative stress, vascular abnormalities, neuronal, and glial cell death
Arun et al. (137)	Mouse/A compressed air-driven shock tube/21 psi	6 or 24 h	Three blast (1.5 min)	Mice restrained in the prone position with a tautly-drawn net		Initial decrease and later increase GFAP and total tau proteins (liver, spleen, brain, and plasma)
Zou et al. (138)	Rat/5 kg TNT and PETN detonation: 3 m distance (high exposure, 480 kPa) and 2 m distance (low injury, 180 kPa)	24, 72 h and 2 weeks	Single	None		Retina injury: blast-dependent increase in VEGF, iNOS, eNOS, nNOS, AQP4, GFAP, elevated inflm cytokines, and chemokines
Prima et al. (139)	Rat/composite blast with head acceleration and Primary blast with no acceleration/230–380 kPa	6 h and 1 and 7 days	Single	Primary blast vs. composite’ blast animals are body armored		Thrombin generation (TG) serum integrin α/β, sE-selectin, sICAM-1, and matrix metalloproteinases MMP-2, MMP-8, and MMP-13
Tumer et al. (104)	Rat/compressed air-driven shock tube ~2 m distance/358 kPa for 10 ms/noise level noise level (100–105 dB)	6 h	Single	None		Increased oxidative stress; activation of the sympatho-adrenal medullary axis; (TH), dopamine-β hydroxylase (DβH), neuropeptide Y (NPY) plasma norepinephrine (NE); diffused neuronal injury
Genovese et al. (135)	SD-rat/shock tube airblast exposure 74.5 kPa	Every 7 days for 8 weeks	1/day for 3 days	None	Conditioned fear/PTSD	Neuronal pathology
Huber et al. (131)	Mouse/compressed gas-driven shock tube	24 and 30 days	Single	None		Elevation of multiple phospho-, cleaved-tau, and (MnSOD or SOD2) levels
Sajja et al. (140)	Rat/helium shock tube/117 kPa	7.5 ms	24, 48 h	Magic angle spinning 1H MRS analysis		Elevated *N*-acetyl aspartate, glutamate, and increased GFAP, Bcl-2, Bax, caspase-3, signs excitotoxicity (glutamate/creatine; hippocampal neuronal loss; mitochondrial distress
Skotak et al. (141)	Rat/helium driven shock tube/(130, 190, 230, 250, and 290 kPa)	24 h	Single	Biomechanical loading assessed with pressure gauges (thorax, cranial space, and nose)		Diffuse blood-brain barrier breakdown in brain parenchyma; fatality; lung hemorrhage; no evident neuronal injury
Valiyaveettil et al. (34)	Mouse/blast overpressure/20.6 psi	4, 24, and 72 h	Three times (1–30 min)	None		Platelet serotonin decreased at 4 h post blast; increase in the plasma serotonin levels. Increase in blood, plasma, and brain myeloperoxidase enzyme activity. Constriction of blood vessels of the brain
Takeuchi et al. (142)	Rats/laser-induced shock waves/0.5–1, 0.5 J/cm^2^	14 days	Single	None		Decrease in the CB (cingulum bundle) axonal density
Turner et al. (143)	Rats/tabletop shock tube/31, 50, 72, and 90 psi	72 h	Single	Thorax and abdomen protection		Neural degeneration; increased glial activation (GFAP); extensive intracranial bleeding leading to death
Tweedie et al. (144)	Mouse/concussive head trauma (weight drop with metal protection)/explosion shock wave pressure (7 m distance ~2.5 psi–17.2 kPa)	7 days	Single	Comparison between mild TBI and blast injury	Altered cognitive and emotional behaviors (Y maze, novel object recognition passive avoidance/elevated plus maze cognition and anxiety	Altered hippocampal gene expression
Cho et al. (134)	Mouse/bast chamber (compression wave attached to a PVC tube)/94, 123, and 181 kPa	7, 14, 28 days and 3 months	Single	Body is protected with fiberglass screen mesh/hearing loss model		Decreased spiral ganglion neurons (SGNs) and afferent nerve synapses, loss of outer hair cells (OHCs), tinnitus, hearing loss
Yeoh et al. (103)	SD rat, rifle primary shock tube (145, 232, and 323 kPa)	5 min and 24, 48 h	Single	None		IgG assessment cardiovascular injury due to primary blast injury is distinct from a typical TBI
Cho et al. (134)	Male SD rat, shock tube 129.23 ± 3.01 kPa for 2.5 ms	4, 24, 48 h and 2 weeks post BOP	Single	None	Short term memory	Immunological assessment (TMF-γ, MCP-1) neuronal loss
Ahlers et al. (145)	Rat/pneumatically driven shock tube at 116.7, 74.5, and 36.6 kPa	6, 24 h and 1 week	Single or 12 blasts (24 h at 36.6 kPa)	Three body orientation (sideway, facing away vs. frontal)	Morris water maze task 116.7 kPa demonstrated transient alteration or loss of consciousness, 74.5 kPa demonstrated anterograde memory deficits	Subdural hemorrhage and cortical contusions
Ahmed et al. (146)	Swine/blast overpressure/mild (24–37 psi) or moderate (40–52 psi)	6, 24, 72 h and 2 weeks	Single	None		CSF biomarkers (CK-BB NFH, GFAP, S100B, VEGF, Claudin 5, and NSE); neuronal and glial cell damage, altered vascular permeability, and inflammation
Balakathiresan et al. (123)	Rat/air-driven shock tube 120 kPa	3 and 24 h	Short interval (three times – 2 h), long interval (three times – 24 h each)	None		CSF and serum miRNAs (let-7i)
Hines-Beard et al. (147)	Mouse/primary ocular blast injury; pressurized air tank with paintball gun/23.6, 26.4, and 30.4 psi)	3,7, 14, and 28 days		Visual acuity deficit detected in 30 psi group eyes via optokinetics		Retinal damage was present in the eyes from the 30 psi group-corneal edema, corneal abrasions, at optic nerve avulsion
Bir et al. (148)	Rat/gas-driven shock tube, 90, 103, 117, 193, and 159 kPa	24, 48, and 72 h	Single	None		MRI analysis showed hippocampal reduction in the Cerebral Blood Flow
Kovesdi et al. (150)	Rat/shock tube/20.6 psi	8 and 45 days	Single	Minocycline (50 mg/kg i.p. NSAID); mitigate neurobehavioral changes/body protection	Impaired memory and increased anxiety. (open field, elevated plus maze, and Barnes maze) minocycline showed neuroprotection	Elevated brain and Serum: CRP, MCP-1, NFH, NSE, Tau, GFAP, MBP, S100B, CRP, MCP-1, TLR-9, Claudin 5, and AQP4
Li et al. (95)	Macaca fascicularis/120 kg of TNT/80 and 200 kPa	3 days and 1 month	Single and double (3 days interval at 80 kPa)	Monkey Cambridge neuropsychological test automated battery motor coordination and working memory		Increased (AQP-4) white matter degeneration, astrocyte hypertrophy; MRI revealed ultrastructural in Purkinje neurons in the cerebellum and hippocampal pyramidal neurons
Rafaels et al. (51)	Ferrets/8′shock tube/variable peak overpressure (98–818 kPa range)	1–5 h	Direct recording	Head exposure/thorax and abdomen protection		Apnea; brain bleeding; fatality
Shridharani et al. (153)	Pigs/compressed-gas shock tube/variable (107–740 kPa range)	1.3–6.9 ms	Direct recording	Heads exposed/lungs and thorax protected (ballistic protective vests)		Apnea intracranial pressures indicates pressure attenuation by the skull up to a factor of 8.4
Sundaramurthy et al. (96)	Rat/Nebraska’s shock tube/100, 150, 200, and 225 kPa)	NA	Single	Variable *Animal Placement Location* along the shock tube (i.e., inside, outside, and near the exit)		Surface and intracranial pressure elevation linearly with the incident peak overpressures
Svetlov et al. (92)	Rat, external shock tube (230–380 kPa)	1 and 7 days post trauma	Single	Primary and composite blast		Persistent gliosis accumulation of GFAP/CNPase in circulation as well as IL-1/IL-10 fractalkine, orexin A, VEGF-R, NRP-2 increased after primary, and composite; integrin-α/β, ICAM-1, L-selectin, NGF-β increased after primary blast
Elder et al. (154)	Rat/air blast shock tube (WRAIR)/74.5	4.5 months	Three times (24 h)	Anxiety and fear; locomotor activity, MWM, rotarod, elevated zero arm, predator scent exposure; movement restricted with shielding; contextual and cued fear conditioning		Elevation in the amygdala of the protein stathmin 1 (proteomic changes)
Dalle Lucca et al. (155)	Rat/compressed air-driven shock tube/120 kPa	0.5, 3, 48, 72, 120, and 168 h	Two	None		Hemorrhage and edema in the brain cortex; elevated TNF-α, C3/C5b-9, and AQP-4; increased leukocyte infiltration
Arun et al. (22)	*In-vitro* 96 well plates-SH-SY5Y human neuroblastoma cells bTBI model/compressed air-driven shock tube (13.68, 18.03, and 21.05 psi)	24 h	Sing1e or three times (2 min intervals at 21.05 psi)	Plate orientation (horizontal vs. vertical)		Decreased ATP levels, increased LDH, and ROS; downregulation of CyPA protein
Chavko et al. (62)	Rat/air-driven shock tube/36 kPa point-pressure measurements of cerebral ventricles	~2.94 ms	Single	Head orientation (head facing blast, right side exposed, head facing away)		Pressure wave propagation and head orientation dependence
Kuehn et al. (156)	Rat/cranium only blast injury apparatus/137.9–515 kPa	24 h and 7 and 10 days	Single	None	Accelerating rotarod; apnea	H&E staining subarachnoid hemorrhages; brain injury (caspase-3, and β-amyloid precursor protein (β-APP), IgG labeling, and Fluoro-Jade C); cardiac arrest; vasogenic edema
Cernak et al. (157)	Mouse/helium modular, multi-chamber shock tube/mild (183 kPa) moderate (213 kPa), severe (295 kPa)	1–5, 7, 10, 14, 21, and 30 days	Single	Supine vs. prone position)	Motor, cognitive, and behavioral) outcomes, assessed via : rotarod, anxiety learning, and memory via active avoidance procedure	Inflammation elevated in tissue CCL, osteopontin, MRP8, ED1, and GFAP at different time points
Koliatsos et al. (50)	Mouse/helium multi chamber shock tube/high (25–45 psi), low (2.1 psi)	3, 5 days (biochem testing) and 7–14 (behavioral)	Single	Either Head or Torso Covered	Rotarod, Y maze open field social and spatial recognition memory and motor deficits	Axonal swellings (injury), APP, but degeneration staining 7–14 days after exposure
Kovesdi et al. (149)	Rat/compression-driven shock tube/20.6 psi	15, 44, 66 days (behavioral) and 66 days (biochemical)	Single	Enriched environment (EEN) contribution	Memory problems, increased anxiety, and depression; improved spatial memory in EEN	Axonal degeneration; elevation in IL-6, IFNγ VEGF, and tau protein levels; hippocampal GFAP and DCX
de Lanerolle et al. (53)	Swine/explosive blast levels in three scenarios: simulated free field (35 psi), high-mobility, vehicle (65 psi), and building setup (63 psi)	72 h and 2 weeks	Single	Blast varied settings: blast tube, high mobility; multipurpose wheeled vehicle, and four-sided structure		Little neuronal injury, fiber tract demyelination, or intracranial hemorrhage observed; increased astrocyte activation; bulbs positive for BAPP
Pun et al. (47)	Rat/120 kg of 2,4,6-trinitrotoluene (TNT)/48.9 kPa (7.1 psi) or 77.3 kPa (11.3 psi) at 24 or 40 m	1, 4, and 7 days	Single	Concrete block was placed between the animals and the explosive source at a distance of 1.5 m from the animals		Cortical neurons were “darkened” and shrunken with narrowed vasculature (day 1, not at 4–7 days); no Iba-1 change; TUNEL-positive cells in the white matter of the brain (day 1); an increase in APP in the white (acute axonal damage); genomics analysis showed signs of repair at day 4 and 7 post-blast
Reneer et al. (151)	Rat/multi-mode shock tube, the McMillan blast device (compressed air/helium driven tube mode, or oxyhydrogen – RDX explosives mode/ 100, 150, and 200 kPa)	3 min post blast	Single	Two overpressure modes (air vs. explosives), Kevlar vest body protection		Rats exposed to compressed air-driven blasts had more pronounced vascular damage than those exposed to oxyhydrogen-driven blasts of the same peak overpressure
Risling et al. (152)	Rat/blast tube with pressure wave/130 and 260 kPa	2 h, 1, 3, 5 days, and 3 weeks		Three groups comparison – (1) fixed no head acceleration forces; (2) controlled penetration of a 2-mm thick needle; and (3) high-speed sagittal rotation angular acceleration		Diffuse axonal injury (DAI) in penetration and rotation models; genomics changes in the expression in a large number of gene families cell death, inflammation, and neurotransmitters in the hippocampus (acceleration and penetration injuries); downregulation of genes involved in neurogenesis and synaptic transmission
Rubovitch et al. (93)	Mouse/open field explosives ~500 g TNT detonation (1 m elevated)/5.5 and 2.5 psi	30 days		Mice in plastic net 4 or 7 m; MRI and DTI analysis	Significant decrease in cognitive and behavioral (Y maze; hippocampal function and spatial memory; novel object recognition task	Increased BBB permeability; 1 month post-blast; increase in fractional anisotropy (FA); no visible organ damage; and elevated MnSOD2
Connell et al. (158)	Female Guinea pig/2.5-cm strips of shock tubing/(23, 41, and 64 kPa	30 min		*Ex vivo* model of spinal cord white; shock tubing (explosive lining of 0.1 grain/foot composed of tetranitramine and aluminum)		Nervous tissue compression, and increased axonal permeability
Garman et al. (54)	Rat/helium-driven shock tube/35 psi (4 ms)	24, 72 h and 2 week		Head exposure with body armor		Increased blood–brain barrier permeability; elevated APP, GFAP, Iba1, ED1, and rat IgG.
Gyorgy et al. (122)	Pig/compression-driven shock tube/~20, 20–40, and ~40 psi	6, 24, 72 h and 2 week		None		Serum elevation of S100B, MBP, and NF-H, but not NSE
Readnower et al. (44)	Rat/air-driven shock tube/120 kPa	3, 24 h and 5 days	Single	None	BBB breakdown: At 3 and 24 h post exposure; increase in IgG staining in the cortex; brain oxidative stress: (4-HNE) and (3-NT) were significantly increased at 3 h post exposure and returned to control levels at 24 h post exposure; and microglia activation: at 5 days	
Cheng et al. (159)	Rat/electric detonator with the explosive equivalent of 400 mg TNT (100, −400 kPa) (distance of 5, 7.5, and 10 cm)	1, 2, 3, 5, and 7 days	Single	Head orientation(frontal, parietal, and occipital head exposure)	87% Rats developed apnea, limb seizure, poor appetite, and limpness	Diffuse subarachnoid hemorrhage and edema; cortical capillary damage; and tissue water and NSE
Cai et al. (160)	Rat/5 g compressed dynamite stick (75 cm from chest)	3, 6, 12 h and 1, 2, 3, 7 days	Single	Blast vs. burn-blast		Serum neutrophil elastase (NE); water lung content
Long et al. (10)	Rat/compression-driven shock tube/126 and 147 kPa	24 h	Single	Kevlar – protective vest (thorax – abdomen)	MWM testing beam walking and spatial navigation(disrupted neurologic neurobehavioral performance)	Heart rate, MAP, brain axonopathy, and widespread fiber degeneration
Säljö et al. (42)	Rat shock tube/10, 30, and 60 kPa (4 ms)	0.5, 3, 6, and 10 h and 1, 2, 3, 5, and 7 days	Single	Morris water maze: impaired cognitive function: 48 h post injury		Dose-dependent rise in intracranial pressure ICP in rats exposed to blast and an increasing time delay in elevation with decreasing intensity of exposure. the ICP returned to control levels after 7 days
Säljö et al. (41)	Pig – Howitzer (9 and 30 kPa); Bazooka (42 kPa); automatic rifle (23 kPa)	3 and 7 days	Three (exposure in air; 15 min intervals) two (exposure under water; 6–7 min)	Comparison of pressure time of different blast overpressure in: air, underwater, and localized blast		In pig study: small parenchymal and subarachnoid hemorrhages, predominately in the occipital lobe, cerebellum, and medulla oblongata; no observation in rat study
	Rat/shock tube (8.7 kPa)	
Cernak et al. (45)	Rat/large-scale BT-I shock tube/3389 kPa and small-scale BT-III shock tube (440 kPa)	3, 24 h and 5 days	Single	Protected head vs. whole body exposure	Deficits in active avoidance task	Swellings of neurons, glial reaction, and myelin debris in the hippocampus, laminal body and vacuoles formation (electron microscope)

*B APP, B-amyloid precursor protein; GFAP, glial fibrillary acidic protein; AQP-4, aquaporin-4; MnSOD or SOD2, manganese superoxide-dismutase l; UCH-L1, ubiquitin C-terminal hydrolase; vWF, von Willebrand factor; NA, not applicable; NSE, neuronspecific enolase; Mwm, Morris water maze; CK-BB, brain-specific creatine kinase; MAP, mean arterial pressure; H&E, hematoxylin and eosin; 4-HNE, 4-hydroxynonenal; 3-NT, 3-nitrotyrosine; TNT, 2,4,6-trinitrotoluene; RDX, oxyhydrogen; ms, milliseconds; MMP8, matrix metalloproteinase 8; BOP, blast over pressure; NF-H, neurofilament-heavy chain*.

Several studies have been performed to assess neuropathological effect of BOP coupled with other comorbid factors (17, 29, 47–51). In these studies, several parameters were varied (different blast injury models, intensity, animal species used) or other modifications were included (protective vests, stressors, and animal localization).

One representative study is that of Kamnaksh et al. where they assessed different stressors and their contribution to blast injury. These stressors included transportation and blast sound with or without blast injury. Of interest, all groups exhibited increased anxiety, while injured and blast noise-exposed rats showed elevated corticosterone, interferon-c (IFN-c), and interleukin-6 (IL-6) in the amygdala and hippocampus. Injured animals showed elevated Iba1, GFAP, and apoptotic immunoreactivity (52). These data demonstrate that exposure to biological stressors can lead to behavioral changes and trigger specific neuropathological alteration even in the absence of detectable injury.

Pun et al. using a rat model, assessed the effects of a single sublethal blast over pressure (BOP) exposure (48.9–77.3 kPa) in an open-field set up. Histopathological analysis of inflicted brains revealed “darkened” and shrunken cortical neurons with narrowed vasculature at day 1 post-injury. Signs of recovery were demonstrated at days 4 and 7 post-blast exposure. Oligodendrocytes and astrocytes showed TUNEL-positivity in the white matter at day 1. Acute axonal damage was observed in the white matter as indicated by elevated amyloid precursor protein immunoreactivity with no sign of macrophages/microglia change. Major gene changes were observed at day 1 and 4 post-blast pointing toward signs of repair at day 4 and 7. These findings suggest that the BOP levels in the study resulted in mild cellular injury and white matter perturbations (47). In another study by Koliatsos et al. primary (BOP) wave effect of mild BOP (68, 103, and 183 kPag) was compared to secondary and tertiary effects. Using a shock tube generating shock waves, the effects of blast on parenchymatous organs including brain, were evaluated. The main injuries in non-brain organs included hemorrhages in the lung interstitium, hemorrhagic infarcts in liver, spleen, and kidney. Neuropathological changes and behavioral outcomes were evaluated at mild blast intensity showing signs of multifocal axonal injury in the cerebellum, the corticospinal system, and optic tract. These findings were accompanied with prolonged behavioral and motor abnormalities (deficits in social recognition, spatial memory, and in motor coordination). Interestingly, shielding of the torso ameliorated axonal injury and behavioral deficits (50).

In a different study, de Lanerolle et al. used a swine model to assess different scenarios of blast exposure including: simulated free field (blast tube), high-mobility multipurpose wheeled vehicle surrogate, and building 4-walled structure. Of interest, histological changes in the three blast scenarios showed minimal neuronal injury with fiber tract demyelination and intra-cranial hemorrhage. Neuropathological changes involving increased astrocyte activation coupled with proliferation and periventricular axonal injury detected were observed with β-amyloid precursor protein (53).

Long et al. assessed blast-induced physiological, neuropathological, and neurobehavioral changes coupled with Kevlar protective vest encasing the thorax and part of the abdomen using a compression-driven shock tube (at 126- and 147-kPa). Kevlar vest effect reduced air blast mortality and also ameliorated the widespread fiber degeneration in rat brains. BOP was shown to induce abnormal neurologic and neurobehavioral performance along with cardiovascular disruptions involving hemorrhagic hypotension with disruption in cardio-compensatory resilience (reduced peak shed blood volume, etc.) (10). Similarly, Rafaels et al. using a male ferrets with protected thorax and abdomen, evaluated intra-cranial hemorrhage and cardiorespiratory coupling at different ranges of blast exposures. Increasing severity of blast exposure demonstrated increasing apnea immediately after blast accompanied by hemorrhages in proximity to the brain stem (51).

In an interesting study, Garman et al. characterized the neuropathological changes produced by a single blast exposure in rats with body shielding using a helium-driven shock tube (exposure of 35 Psi with left side-head-only exposure) (54). Neuropathological analysis was conducted at various time points (24 h, 72 h, or 2 weeks post-blast). Multifocal axonal degeneration was present in all blast-exposed rats at all-time points coupled with diffused axonal injury in the cerebellar and brainstem white matter tracts. In addition, reactive microglial activation was also identified despite subtle GFAP, ED1, and Iba1 staining. Finally, increased blood–brain barrier (BBB) permeability was seen at 24 h. Findings from this study indicated axonal, dendritic, neuronal, and synaptic degeneration in the initial 2 weeks post exposure with body shielding. Over time, there was also evidence of progression of the axonal degenerative process characterized by increased axonal fragmentation similar to the process of DAI that follows TBI which is suggestive of a therapeutic window in the immediate post-blast period (54).

In conclusion, these different blast studies presented distinguished heterogeneous results (summarized in Table 1); and provided different insights into the associated neuropathological changes occurring post-blast exposure. These findings highlight the challenges encountered in modeling experimental blast injury and translating the findings into preclinical brain injury studies to be evaluated and verified clinically (discussed in different sections).

## Neuronal Injury Mechanisms

The exact mechanism by which BOP mediates neuronal injury has not been fully elucidated (47). The neuropathological changes evoked by BOP are different than those described following acute models of brain injury (i.e., acceleration–deceleration injury or direct impact) (10, 55–58) highlighting at the complex pathways involved. Elegant work with experimental data by Cernak et al. has shown that primary closed non-impact blast injury-induced neurotrauma involves the interaction of cerebral, local, and systemic responses (31, 32, 45, 48). These experimental data seem to highlight the fact that blood vessels vasculature (venous as well as arterial) may be acting as a conduit for blast energy transfer to the brain contributing to blast pressure-induced fiber degeneration.

In non-blast brain injury, the primary injury occurs as a consequence of mechanical force due to direct contusion of the brain against skull’s rough interior or due to shearing and stretching forces against the brain tissue (31, 59). This may also involve vascular injury including subdural hematoma from ruptured blood, brain edema from elevated permeability of cerebral vasculature along with reduced blood flow due to intra-cranial pressure or infarction (59). Taken together, these complications represent the secondary and tertiary phases of blast injury.

Cernak et al. assessed the contribution of body-central nervous system (CNS) cross talk involved in blast-induced trauma related to the activation of autonomous nervous system and the neuroendocrine–immune system which contributes significantly to the mechanism of blast injury. Inflammation has been proposed to play an important role in the pathogenesis of long-term neurological deficits due to blast (31). Experiments using rigid body- or head-protection in animals subjected to blast showed that head protection failed to prevent inflammation in the brain while body protection was able to alleviate blast-induced brain functional impairments highlighting the role of body-CNS interaction (31).

Cernak et al. studies have demonstrated that blast exposure (mild-to-moderate) induces the activation of autonomous nervous system in rabbit exposed to BOP. Distinct pathological components in the brain including impaired energy metabolism, and increase in the sodium–potassium ATPase measured in the brainstem and erythrocyte membranes were coupled with edema formation (48, 60). In addition, to link systemic alteration and cerebral inflammation to long-term neurological deficits caused by blast, migration, and accumulation of polymorphonuclear leukocytes as key inflammatory markers of host response were assessed after helium-driven shock tube delivering mild blast injury (103 kPa). *In vivo* real time imaging of myeloperoxidase (MPO) inflammatory enzyme activity of activated phagocytes was conducted on three groups of rats: (1) whole-body blast; (2) blast with “body armor,” (chest and abdomen) with the head exposed; or (3) blast with “helmet” as head protection (neck and skull) while the rest of the body exposed. One day post-blast exposure, MPO activity was observed in the gastrointestinal tract and the diaphragmal mediastinal parts of the lungs (61).

In the brain, this activity was observed at 7, 14, and 30 days post-blast injury. Of interest, MPO increase in the brain was independent of head protection at 14 and 30 days post-injury suggesting chronic inflammation and highlighting the role of systemic origin of the inflammatory activation mediating brain injury which highly reflects on the role of the vagal afferent neurons mediating gut–brain communication. Taken together, the results of this study clearly demonstrate the importance of the indirect, i.e., blast–body interaction as well as the decisive role of autonomous nervous–neuroendocrine–immune systems interaction in the pathogenesis of blast-induced brain trauma (31).

Similarly, Chavko et al. assessed the theory of the indirect effect of kinetic energy transfer via the blood vessels and the surrounding cerebrospinal fluid (CSF) to the CNS (62). In their work, they evaluated the contribution of direct versus indirect transfer and its correlation to the head orientation and the surface area exposed. Brain biomechanical responses involving pressure inside the brains were assessed in rats exposed to low blast exposure (35 kPa) and positioned in three different orientations with respect to primary blast wave. These positions included: frontal exposure (i.e., head facing blast) right side exposed and head positioned away from blast. Frontal exposures showed higher traces of pressure amplitude and longer duration, suggestive of dynamic pressure transfer (62). On the other hand, the pressure wave inside the brain in the head facing away was similar to hydrodynamic pressure within the brain. It has become more evident that the primary pressure wave can induce functional, biochemical, and morphological alterations in different ways than those observed in other types of traumatic injuries (penetrating head injury).

## Mild TBI and Neuropsychiatric Impairments in Blast Injury and PTSD Comorbidity

Another significant aspect of blast injury is psychological health which is highly affected. Many injured troops returning from war zones are afflicted with blast-induced BI experiencing post concussive symptoms (PCS), characterized by memory and cognitive disruption, irritability, anxiety, and fatigue (63). Among these with mTBI, PCS can persist long after exposure leading to major functional impairments (64). Unlike casualties suffered from moderate to severe TBI patients diagnosed with mTBI present with no apparent structural injury and are conscious with typical symptoms including headache, confusion, dizziness, memory impairment, and behavioral changes.

The nomenclature of mTBI has been a challenge for both civilian and military settings as described by Rosenfeld et al. (65). mTBI, according to the DoD, involves head trauma with loss of consciousness for <30 min or exhibiting post-traumatic amnesia for <24 h (66). Patients with mTBI have a Glasgow coma score of 13–15 usually experiencing poor unspecific diagnostic symptoms involving headaches, cognitive dysfunction, etc. independent whether mTBI is blast related or not. It is of high interest to deliver accurate diagnosis for such condition due to the overlapping symptoms mistaken with neuropsychiatric disorders. This in contrary to the moderate and severe blast-related TBI which have 9–12 and 3–8 Glasgow coma score respectively and require special treatment as they exhibit intra-cranial hemorrhage and brain edema (2, 67, 68). Patients with blast-related severe TBI are characterized with delayed vasospasm, and pseudoaneurysm formation requiring early intervention (2, 67). Severe blast-related TBI cases are usually due to the primary and secondary (penetrating injury) phases of blast and would require strict clinical guidelines that are similar to those in non-blast-related severe TBI cases (65).

Mild traumatic brain injury is the most frequent form of brain trauma among deployed military populations (69). It has been shown that repeated exposure to multiple low levels of blast injury account for the majority of mTBIs cases. These victims remain conscious and often are redeployed without proper diagnosis and treatment while they undergo severe mental stress (70, 71). The heterogeneous presentation of BOB injuries among mTBI patients depends on several factors (similar to what is observed in experimental blast injury studies) including: device composition, environment (e.g., presence of intervening protective barriers), distance from blast, and the use of protective shields, etc. (11, 72).

Primary blast component of blast injury is among the main contributors in developing neuropsychiatric impairments associated with the primary phase profile (30, 73). There had been an urgent quest to for future research examining the impact of blast concussion (particularly recurrent concussion) on neuropsychological performance. Neuropsychological evaluation of cognitive status post-blast exposure can be challenging for a variety of reasons. In particular, clinicians may have difficulty assessing: true concussion severity due to limited knowledge of the blast events which may be reflective of self-report months or years post the event(s) occurrence. In addition, the lack of several features of the blast environment may complicate the accuracy of the “blast self-report” involving distance from the blast, concussion severity which these are often unavailable from primary records (74). Thus, the lack of reliable information pertaining to injury characteristics makes it challenging to determine the course of cognitive recovery and rehabilitation. Usually, concussion severity is usually determined based on current PCS on screening instruments which are not necessarily specific to concussion and can be shared with depression or PTSD or even these PCS may be reflective of PTSD itself as elegantly discussed by Nelson et al. (74). Of interest, Hoge et al. reported that more than 40% of soldiers who experienced symptoms associated with mTBI (loss of consciousness) met the criteria for PTSD (1). This same study suggested that increased rates of health problems reported by soldiers exposed to mTBI are mediated mainly via neuropsychiatric disorders such as PTSD or depression, rather than mTBI (1).

Post-traumatic stress disorder, a psychiatric condition that arises after exposure to a life threatening experience such as conditions experienced in combat war zone with or without blast exposure as a form of mTBI (75). This, by itself, poses a challenge in the clinical diagnosis in veterans who are exposed to mTBI since the symptoms may overlap between these conditions exacerbated by other comorbid conditions such as drug abuse or other neuropsychiatric complications (75, 76). A Rand Corporation study indicated that ~20% of returning service personnel (~300,000) have had a TBI and that there was substantial overlap of TBI with the occurrence of PTSD (77).

Psychological stress resulting from exposure to blast wave leads to an altered psychological health status which contribute significantly to the development of PTSD (52, 70). However, a major recurring question arises-due to the similarity of blast injury clinical symptoms and those of PTSD, is how do we clinically differentiate between these two conditions and other neuropsychiatric conditions?

Post-traumatic stress disorder is deemed an effect of psychological and emotional determinants/trauma (i.e., event associated with threat of harm or loss of life to which the individual responds with extreme fear or horror), while mild bTBI is a result of destructive biomechanical forces acting on the brain (78). There is substantial overlap in symptom profile associated with these two conditions (1). For instance, impaired concentration, increased irritability, insomnia, and lack of interest are among the symptoms shared in the diagnosis for mTBI and PTSD (79). Additionally, blast TBI is a well-documented risk factor for the development of PTSD (80–82). The association between the two conditions is further supported by structural and functional neuroimaging studies showing similar abnormalities in patients with blast-related mTBI as well as in those with PTSD (83–86).Such overlap and link determines and contributes to several ambiguities emphasizing the urgent need for finding reliable objective test to make an accurate diagnosis and to improve the understanding of the nature of the interaction and pathophysiology of PTSD and mild bTBI.

Clinical evaluation of a blast-exposed personnel can be challenging as symptoms may range from neurologic problems, psychiatric, or emotional difficulties which may be attributed to blast or due to other psychiatric disorder where in several instances the occurrence of TBI and PTSD may be suggested (81, 87). For neurological assessment in TBI, similar criterion-based methodology to that in PTSD has been used rendering a specific diagnosis to either condition or even to those with both conditions (PTSD or TBI-exposed) uncertain (87–89). Thus, in many cases, clinical diagnosis may result in high rate of inaccurate PTSD diagnosis in persons exposed to TBI (87).

Based on the above, it is of high interest that an accurate detailed knowledge of blast injury biophysics and injury threshold may assist clinicians in better diagnosis (87). This includes expanded neuropsychological studies of blast injury (both experimental and clinical) to identify accurate, specific and sensitive anatomic, pathophysiologic, and behavioral responses to blast injury as discussed by Bass et al. (87). This is complicated by the complex nature of blast injury involving several combinations of primary or other phases of blast injury (secondary, tertiary, and/or quaternary blast).

## Animal Models of Blast Injury

Over the last several decades, a number of experimental animal models have been implemented to study the mechanisms of blast wave impact which included rats, mice, ferrets, rabbits, and larger animals involving sheep and swine (33, 90–97). These experimental models exhibited heterogeneous outcomes and even contradictory findings which have been attributed to several factors. A summary of the recent and major blast injury studies (2001, 2009–2013) is summarized in Table 1. In addition, there is a lack in the reproducibility of blast injury models and a need to develop blast injury generators that precisely control blast injury parameters similar to other well-defined acute brain injury models such as (controlled cortical impact (CCI) and the fluid percussion (FP) which have been well characterized with predictable neurological, histological, physiological, and behavioral outcomes. Thus, the need of establishing well characterized reproducible models (animal and blast framework) is vital to identify relevant pathogenic pathways involved that can assist in the development of effective diagnostic, prognostic blast specific-biomarkers (panel of biomarkers) (98). Several blast injury instrumentations are available which include: compressed gas-driven shock tubes which are driven by air, helium, or nitrogen gas which may result in unrealistic duration of the overpressure wave leading to an in-appropriate scaling between species (humans and animal models; Table 1) (99).

## Challenges in Animal Models of Blast Injury

There are limited available basic and translational studies relevant to the mechanisms of primary blast-induced brain injury. A better understanding of injury mechanisms is required for the development of protection and treatment options and biomarker identification for prognosis.

**Figure 1:**
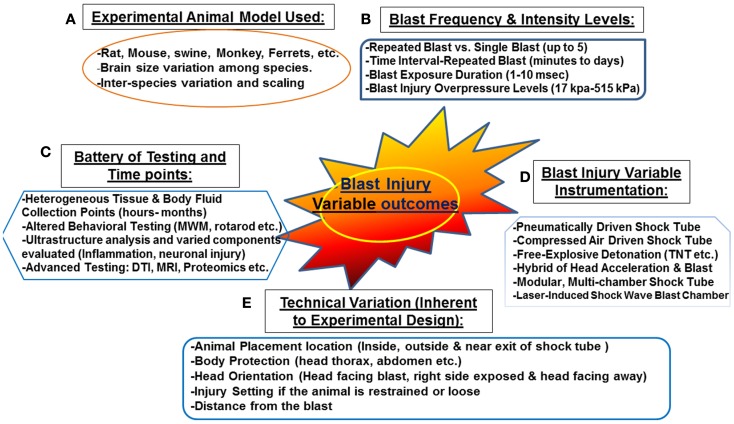
**Challenges associated with “experimental blast injury” modeling real life blast exposure**. Several factors contribute to the heterogeneous behavioral, neuropathological, and systemic profile observed in the several experimental blast injury models. Even with models using the same injury parameters (animal model, blast shock tube, and intensity levels, etc.); reproducing the same results is rather challenging (refer to Table 1). These challenging variables are summarized in the following: **(A)** various animal models and interspecies variation, **(B)** blast injury frequency and intensity levels ranging from single blast up to five blast with some overpressure intensities reaching 515 kPa **(C)** the heterogeneous selection of biochemical/behavioral testing conducted and the several time points selected (hours to few months) **(D)** the non-standardized blast and not well characterized blast injury instrumentation **(E)** technical variation inherent to experimental design related to animal setting, body armor, head protection, and the distance from the blast. These factors contribute to the variable outcome observed in published work in blast injury field.

Several animal models have been proposed at translating intra-cranial biophysics and pathophysiology experienced in human blast exposure (87). These models have a number of limitations including: neuronal tissue biomechanical properties, anatomical differences as well as physiological differences (87). In addition, other factors that are challenging for proper scaling between experimental and human blast injury are associated with neuroanatomy and physiology involving: size of different brain structures, neural mass (brain size, head, body, position, and architecture), as well as body fluid composition (thickness, volume, and components) (87). Other key factors that need to be considered are the potential for exposure scaling, consistency in experimental protocols, frequency of exposure, and overpressure levels, which should be mimicking real life exposure or at least translate equally to human exposure (Figure 1). Other external factors include: distance from the blast, the use of protective shields and the presence or absence of noise stressors, etc. (12) (Figure 1). In real life situation, soldiers are often deployed several times and exposed to numerous psychological stressors such as blast noise with or without blast injury (87). Such conditions can induce adverse physiological changes leading to post-traumatic symptoms without sustaining any prior physical injury (discussed previously). Taken together, these challenging factors contribute to the difficulty of truly modeling blast injury in animals resulting in an in-appropriate neuropathological and neurobehavioral assessment.

## Blood–Brain Barrier and Secondary Injury in Blast Overpressure

Traumatic brain injury leads to progressive pathophysiological changes resulting in a reduction in cerebral blood flow and a decrease in tissue oxygen levels leading to ischemia, BBB disruption with brain edema (100). Death of resident cells of the CNS has traditionally been accepted to take place in two phases: an early necrotic and an on-going long-term apoptotic phase. Secondary brain injury develops in minutes to months following the original insult, progressively contributing to the worsened neurological impairment. This complex phenomenon is defined by the activation of various neurochemical cascades and the systemic physiological responses which manifest following the traumatic event (101).

At the cellular level, the biphasic nature of secondary injury is mediated by numerous disturbed pathways which include: (a) excitotoxicity caused by an excess of the neurotransmitter glutamate; (b) free radical generation by mitochondrial dysfunction, causing damage to proteins and phospholipid membranes of neurons and glia; and (c) the neuroinflammatory response which takes place due to both CNS and systemic immunoactivation. Thus, diffuse brain injury mediated immune responses, BBB alterations, and neuroinflammation seem to play an important role in the pathology of BOP. The increase in BBB permeability was shown to recover by the third day after the blast exposure (44, 102). Following blast injury, loosening of the vasculature and perivascular unit is mediated by the activation of matrix metalloproteinases and water channel aquaporin-4, promoting edema, enhanced leakiness of the BBB, and progression of neuroinflammation and neuronal degeneration (102). Although many studies demonstrate a similar pathophysiologic progression as the conventional TBI, a recent study reported that cerebrovascular injury due to primary blast is distinct from it; suggesting that BBB disruption in blast injury was an acute one, not resulting from a delayed inflammation as it is in the conventional ones (103).

Recent work from our laboratory has shown that blast injury leads to oxidative stress and autonomic dysfunction (104). Generation of free radicals and hypoxia leads to the failure of the Na^+^, K^+^-ATPase, a membrane-bound enzyme required for cellular transport. Dysfunction of this pump is a common feature in CNS pathologies related to ischemic conditions and TBI. The activity of Na^+^, K^+^-ATPase pump is very sensitive to free radical reactions and lipid peroxidation. Reductions in this activity can indicate membrane damage indirectly. Thus, Na^+^, K^+^-ATPase is clearly down regulated under low O_2_ conditions which in turn triggers brain edema, enhances the loosening of tight junctions and causes BBB breakdown. MPO activity, an index for neutrophil infiltration, also increases as an evidence of inflammation (105). In summary, failure of pumps, cerebral edema, BBB permeability, neuroinflammation, and oxidative damage are among the major mechanisms that play important roles in the development of secondary brain injury following TBI.

## Traumatic Brain Injury and Autonomic Dysfunction

One deleterious consequence of brain injury is autonomic nervous system dysregulation and/or dysautonomia. Autonomic nervous system dysfunction has been documented after TBI but is not well understood. Ninety percent of TBI patients demonstrate signs of autonomic dysfunction during the first week after injury, with about one third of the patients developing longer lasting autonomic dysfunction. Autonomic dysregulation is characterized by distinct changes in cardiovascular hyperactivity, sleep function, and specific biomarkers of neural damage. System dysregulation might lead to a range of comorbidities such as hypertension, endothelial dysfunction, and end-organ perfusion abnormalities. Specifically, TBI disruption of autonomic function most often results in sustained sympatho-activation. This sympathetic hyperactivity after TBI remains poorly understood, although sympathetic hyperactivity likely contributes to the high morbidity and mortality associated with TBI. Sympathetic hyperactivity contributes to systemic stress, including neuroinflammation and oxidative stress in the autonomic nervous system. Eventually these disturbances lead to cardiovascular dysfunction (31, 32, 106) and sleep complications (107). Systemic stress is associated with activation of the hypothalamic-pituitary-adrenal (HPA) axis (108) and the hypothalamic sympatho-adrenal medullary axis (109). It is known that TBI activates the HPA, however little is known regarding the TBI-induced activation of the sympatho-adrenal medullary axis, and there are limited therapeutic options to treat this sympatho-activation.

We recently demonstrated selective biochemical markers of autonomic function and oxidative stress in male Sprague Dawley rats subjected to head-directed overpressure insult (104). There were increased levels of tyrosine hydroxylase (TH), dopamine-β hydroxylase (DβH), Neuropeptide Y (NPY) along with plasma norepinephrine (NE). In addition, blast-induced injury significantly elevated TH in the nucleus tractus solitarius (NTS) of the brain stem while AT1 receptor expression and NADPH oxidase activity, a marker of oxidative stress, was elevated in the hypothalamus suggesting that single BOP exposure results in increased sympatho-excitation. The mechanism may involve the elevated AT1 receptor expression and NADPH oxidase levels in the hypothalamus. Taken together, such effects may be important factors contributing to pathology of brain injury and autonomic dysfunction associated with the clinical profile of patients following BOP exposure (104).

## Blast Brain Injury and Oxidative Stress

The primary effects of BOP have been generally attributed to its external physical impact on the body, causing internal mechanical damage. The pathophysiological effects on hollow organs have been extensively studied, but little attention has been given to the biochemical manifestations and molecular mechanism(s) of injury occurring in the brain after BOP exposure. Due to the biochemical nature of BOP compared to physical nature of TBI (impact or penetrating injury), subtle molecular changes such as free radical-mediated oxidative stress occur and contribute to the manifestation of BOP-induced brain injury (40, 44, 110). Previous studies have demonstrated that reactive oxygen species such as the superoxide radicals and nitric oxide can form peroxynitrite, a powerful oxidant that impairs cerebral vascular function following blast-induced brain injury (46, 111). Cernak et al. reported that bilateral vagotomy successfully mitigated bradycardia, hypotension, and apnea caused by blast; prevented extreme metabolic alterations and brain edema; but failed to eliminate oxidative stress in the brain due to blast (48). More recently, it was reported that the induction of oxidative and nitrosative damage leads to cerebrovascular inflammation in an animal model of mTBI induced by primary blast (102). Brain-specific oxidatively modified protein markers that are indicative of biochemical/proteomic and functional changes occurring post-BOP need to be considered. Insufficient published data are available to describe the long-term effects of TBI on central noradrenergic systems, particularly on neuroplastic adaptations within numerous targets of central noradrenergic projections. In addition, understanding the etiology of these changes may shed new light on the molecular mechanism(s) of injury, potentially offering new strategies for treatment.

## Blast Injury Biomarkers Identification and Limitations

The widespread recognition of the brain vulnerability to blast exposure and inadequate approaches to diagnose blast-related TBI led to design an mTBI Diagnostics Workshop (66) and the foundation of the Demographics and Clinical Assessment Working Group of the International and Interagency Initiative (112) to assess the current diagnostics technologies that can be used to detect brain injury following mTBI and BOP. One of the major recommendations was the use of biomarkers to supplement functional and imaging-based assessments for significant improvements in the diagnosis and characterization of the effects of blast exposure on brain and for distinguishing bTBI from other neuropsychiatric disorders including PTSD.

Current available imaging modalities, such as computed tomography (CT) and magnetic resonance imaging (MRI), primarily detect major structural changes in the brain (113); however, their utility has not been fully optimized following blast-related mTBI. More advanced neuroimaging techniques such as DTI, while have shown abnormalities post-blast-related TBI (114), have not been able to show consistent relationship to mild bTBI diagnosis (115). Additionally, there is no consensus on the ideal scan method or timing. Therefore, multiple studies have been conducted to identify ideal sensitive, inexpensive, non-invasive biochemical markers that can offer diagnostic and prognostic information, and reflect bTBI pathogenic mechanisms and pathology (116, 117).

To date, several biomarkers such as GFAP (118), UCH-L1 (119), and S-100ß(120) have been identified as potential excellent “candidates” of blast TBI. However, a limited number of studies did specifically evaluate biochemical brain damage markers in the setting of blast-induced brain injury (43, 121). In one study by Svetlov et al. they assessed temporal pattern of serum putative biomarkers that have been characterized in acute TBI including GFAP, NSE, and UCH-L1 in brain tissue, CSF, and blood. Serum biomarkers levels distinctively increased 24 h post-blast, followed by a decline thereafter, indicating a potential use to assess blast-induced brain damage acutely after injury (33). Supporting these observations, Gyorgy and colleagues, using reverse phase protein microarray (RPPM) technology to determine serum protein levels, showed a rise in S-100B, MBP, NF-H, and NSE protein levels in serum after injury in a large-animal model of bTBI. Remarkably, serum NF-H was reported to increase in an overpressure dose-dependent manner reflecting the extent of the damage caused by bTBI (122).

More recently, Balakathiresan et al. proposed microRNAs as novel serum diagnostic biomarkers of bTBI. They investigated microRNA signatures in CSF and serum of rats exposed to BOP injury. Specifically, microRNA let-7i was elevated in both CSF and serum post-blast wave exposure and was considered as an ideal candidate biomarker of brain injury (123). Importantly, microRNAs can be considered the third generation molecular signature after proteomics and genomics studies (123). Elevated concentrations of serum vascular endothelial growth factor, associated with neuroinflammation and vascular pathology in blast-related TBI have also been reported (124).

Studies investigating biomarkers of mTBI in humans continue to be limited as illustrated in one study by Ingebrigtsen and Romner (125). In their research paper, MEDLINE database was surveyed for biochemical serum markers specific to mild head injuries. Three serum markers including creatine kinase isoenzyme BB (CKBB), NSE, and S-100B were evaluated. Of these markers, S-100B protein was proposed as the most promising marker for mTBI while the other two lacked specificity, sensitivity, or injury correlation (125). In an another study by Blennow et al. military personnel exposed to explosions or repeated firing of heavy weapons did not show any evidence of brain damage as assessed by CSF biomarkers. (126). Conversely, the New Zealand Breacher Study demonstrated a degree of brain perturbation as assessed by serum biomarker levels, neurocognitive performance, and self-reported symptoms in members of the New Zealand Defense Force exposed to repeated low-level blast (127). Taken the controversial results of these different studies, these findings, in fact, stimulate the need for further research to evaluate the usefulness of biochemical markers after repeated exposure of different blast levels.

Interestingly, recent experimental and human studies are suggesting a link between blast exposure and chronic traumatic encephalopathy (CTE), a tau protein-linked neurodegenerative disease (128–131). To date, no biofluid marker has been shown to assist with diagnosis of CTE. However, future studies to identify biomarkers tracking chronic processes and on-going degeneration and able to predict the development of neurodegenerative diseases of bTBI are of a critical need.

## Future Recommendations

For long, TBI has been considered one of the “signature injuries” of current conflicts in Iraq and Afghanistan which attracted concern from the DoD, Department of Veteran Affairs, and National Institutes of Health, encouraging combined efforts to understand brain injury pathophysiology and identify therapeutics and assess different approaches for rehabilitation platforms as well as deciphering novel blast specific biomarkers (7, 11). Better understanding of the biophysics of blast shock injury and its body propagation to the neural tissue may enhance the development body armor protection. Given the complexity of blast TBI pathobiology, the development of an objective, specific, and quantifiable panel of biomarkers is highly needed for the purpose of providing better monitoring of the real time injury mechanism and progression post-blast exposure (121, 122, 132, 133). An important consideration is that a panel combining different biomarkers be assembled that can establish the nature and severity of the head injury and reflect the contributing pathogenic mechanism(s) of the acute phase as well as the neurodegeneration and recovery (rehabilitative stages). Additionally, the integration of such bTBI diagnostic markers into routine clinical care will require a thorough validation and extensive standardization protocols coupled with well-defined recommendations for immunoassay and different measurement technologies.

A non-trivial and urgent issue in biomarker-panel design will be determining an appropriate instrument platform that is suited to measure these biomarker changes. At present, biomarkers are analyzed in clinical laboratories using closed, high throughput immunoassay analyzers allowing for high performance in terms of accuracy and precision which are suitable for major hospitals. Future recommendation is to focus research on the development of a miniaturized point-of-care (POC) system, which can be transported in the “field” (military and civilian) providing accurate measurements at a reasonable cost with short turnaround time (116).

## Conflict of Interest Statement

Drs. Prima and Svetlov are employees and receive salaries from Banyan Biomarkers, Inc. The other co-authors declare that the research was conducted in the absence of any commercial or financial relationships that could be construed as a potential conflict of interest.
